# Calcium Channel Protein ORAI1 Mediates TGF-β Induced Epithelial-to-Mesenchymal Transition in Colorectal Cancer Cells

**DOI:** 10.3389/fonc.2021.649476

**Published:** 2021-05-12

**Authors:** Qingjie Kang, Xudong Peng, Xiangshu Li, Denghua Hu, Guangxu Wen, Zhengqiang Wei, Baohong Yuan

**Affiliations:** ^1^ Department of Gastrointestinal Surgery, The First Affiliated Hospital of Chongqing Medical University, Chongqing, China; ^2^ Department of Anesthesiology, The First Affiliated Hospital of Chongqing Medical University, Chongqing, China

**Keywords:** ORAI1, SOCE, TGF-β, EMT, colorectal cancer

## Abstract

Accumulating evidence suggested that calcium release-activated calcium modulator 1(ORAI1), a key calcium channel pore-forming protein-mediated store-operated Ca2+ entry (SOCE), is associated with human cancer. However, its role in colorectal cancer (CRC) progression has not been well studied. Epithelial-mesenchymal transition (EMT) is a multistep process that occurs during the progression of cancers and is necessary for metastasis of epithelial cancer. Transforming growth factor-β (TGF-β) is a pleiotropic cytokine that has been shown to induce EMT. In this study, we are aimed at exploring the effects of ORAI1 on TGF-β1-induced EMT process in CRC cells. Herein, we confirmed ORAI1 expression was higher in CRC tissues than in adjacent non-cancerous tissues by using immunohistochemical staining and Western blot analysis. Higher ORAI1 expression was associated with more advanced clinical stage, higher incidence of metastasis and shorter overall survival. We compared ORAI1 expression in SW480 and SW620 cells, two CRC cell lines with the same genetic background, but different metastatic potential. We found ORAI1 expression was significantly higher in SW620 cells which exhibited higher EMT characteristics. Furthermore, knockdown of ORAI1 suppressed the EMT of SW620 Cells. After induced the EMT process in SW480 cells with TGF-β1, we found treatment of TGF-β1 showed a significant increase in cell migration along with the loss of E-cadherin and an increase in N-cadherin and Vimentin protein levels. Also, TGF-β1 treatment increased ORAI1 expression and was closely associated with the increase of SOCE. Silencing ORAI1 significantly suppressed Ca2+ entry, reversed the changes of EMT-relevant marks expression induced by TGF-β1, and inhibited TGF-β1-mediated calpain activation and cell migration. Finally, we blocked SOCE with 2-APB (2-Aminoethyl diphenylborinate), a pharmacological inhibitor. Interestingly, 2-APB and sh-ORAI1 both exhibited similar inhibition effects to the SW480 cells. In conclusion, our results demonstrated that ORAI1 could mediate TGF-β-Induced EMT by promoting Ca2+ entry and calpain activity in Colorectal Cancer Cells.

## Introduction

Colorectal cancer (CRC) is the third most commonly diagnosed cancer in the world. The incidence and mortality show wide geographical variations (1). It has become a predominant type of cancer and now accounts for approximately 10% of cancer-related mortality in western countries (2). And in Asian countries, the incidence of colorectal cancer has experienced an increase of two to four times during the past few decades (3). According to 2015 data from the National Cancer Center, China ranks third in the incidence and fifth in mortality of colorectal cancer (4). A growing number of studies support the notion that metastasis rather than the primary tumor is the principal cause of cancer-associated deaths (5). However, the exact molecular mechanism of tumor metastasis is still poorly understood.

Metastasis is a multi-step process beginning with the invasion of the cancer cells into surrounding tissue, intravasation of cells into the blood stream, extravasation to the secondary site, and finally, regrowth of the tumor cells as secondary metastases (6, 7). For metastasis to occur, a tumor cell shifts from a polarized epithelial state to an invasive mesenchymal phenotype through a process known as Epithelial-to-Mesenchymal Transition (EMT) (8). The EMT morphological changes transform the cell from a stationery form to a motile form, and several studies show that EMT and cell movement are regulated by Ca2+ influx (9, 10). Ca2+ is a multifunctional secondary messenger. Intracellular Ca2+ homeostasis is required for the development and maintenance of many physiological functions, such as movement, secretion, and gene transcription (11). Intracellular Ca2+ imbalance caused by carcinogenesis has been known to deregulate cell proliferation, migration and suppression of apoptosis (12). In non-excitable cells, the predominant Ca2+ influx pathway is store-operated Ca2+ entry (SOCE) (13). Although SOCE was first proposed in 1986 to describe the process that the depletion of intracellular Ca2+ stores causes the influx of extracellular Ca2+ (14), the identity of the channels key molecular components: ORAI1 and stromal interacting molecule 1 (STIM1) were discovered during the last decade (15). ORAI1 is a pore-forming protein located on the plasma membrane and exhibits four transmembrane domains (16), which is activated by the endoplasmic Ca2+ sensor STIM1 upon Ca2+ store depletion, promoting Ca2+ influx for Ca2+ store refilling and the activation of key Ca2+-dependent processes (17, 18).

Interestingly, recent studies have shown that ORAI1 is overexpressed in many types of cancer, such as lung cancer (19), hepatocarcinoma (20), and gastric cancer (21). Clinically, ORAI1 overexpression is strongly linked to poor clinical outcomes in gastric cancer patients (21). Furthermore, the high expression of ORAI1 enhanced cell proliferation and is associated with poor prognosis in colorectal cancer (22). *In vitro*, EMT-like changes of the cellular phenotype were noticed in ORAI1-knockdown gastric cancer cells, EMT mark gene E-cadherin mRNA expression was upregulated by silencing ORAI1 (21). Earlier studies found that ORAI1 is shown to be critical for breast tumor cell migration and metastasis (23). Moreover, ORAI1-mediated SOCE is reported to play an important role in the progression of various cancers (24). SOCE regulates cellular functions by promoting the entry of extracellular Ca2+ to the cytosol and activating Ca2+-dependent proteinases (25). Some studies have shown that blocking SOCE by using a specific blocker or by applying siRNA that target ORAI1 can inhibit the migration, invasion, and cell movement of cancer cells (9, 21, 26). These results suggested that there should be a direct relationship between ORAI1 and EMT, but the internal mechanisms are yet to be fully clarified.

Transforming growth factor-β (TGF-β), a pleiotropic cytokine, is found in a variety of tissues and is important for cell proliferation, differentiation, and apoptosis (27). In the tumor microenvironment, TGF-β is a strong inducer of the EMT. After treatment with TGF-β, the cell undergoes rapid and dynamic changes in gene regulation with morphological and migratory changes (28). TGF-β induces EMT and promotes cell migration and invasion through the downregulation of epithelial factors and upregulation of mesenchymal factors (29, 30). Calcium signaling serves as a bridge to link signals from the tumor microenvironment with specific cellular responses. For example, TGF-β induced EMT in MCF7 breast cancer cells has also been shown to be associated with increased Ca2+ influx into the cell (9). TGF-β-treatment caused an increase in Ca2+-induced calpain activity, which reduced E-cadherin protein levels, thereby increasing cell migration (29). However, the relationship between TGF-β-induced EMT and Ca2+ signaling remains unclear, and the functions of ORAI1-mediated SOCE in the TGF-β-induced EMT in colorectal cancer cells have not been investigated sufficiently.

In this study, we investigated the expression of ORAI1 in CRC tissues and found that ORAI1 overexpression is associated with progression and poor prognosis of CRC. Knockdown of ORAI1 suppressed the EMT of SW620 Cells. Moreover, we explored the role of ORAI1 and SOCE in the TGF-β1-induced EMT of SW480 cells. By knockdown the ORAI1 expression and the use of SOCE inhibitor, we found that ORAI1 and ORAI1-mediated SOCE plays an important role in TGF-β1-induced EMT in SW480 cells. Thus, ORAI1 and SOCE may be candidates for CRC therapy.

## Materials and Methods

### Patients and Clinical Samples

The human colorectal cancer samples (n=80) and corresponding non-tumor tissues for immunohistochemistry were collected from each patient immediately after the surgical process at the First Affiliated Hospital of Chongqing Medical University from January to November 2014. None had received neoadjuvant therapy before surgery. All of the patients had complete clinical and follow-up data for five years. For Western blot expression studies, 40 pairs of fresh samples were frozen in liquid nitrogen immediately after surgical removal and maintained at -80°C until use.

### Cell Culture

The SW480 and SW620 human CRC cell lines were respectively provided by the Cancer Laboratory and Central Laboratory of the First Affiliated Hospital of Chongqing Medical University. These cell lines were routinely maintained in RPMI-1640 medium (HyClone, Logan, UT, USA) supplemented with 10% fetal bovine serum (HyClone, Logan, UT, USA) at 37°C in a humidified air atmosphere containing 5% CO_2_. Before stimulation with TGF-β1 (PeproTech, Rocky Hill, NJ, USA), cells were cultured with medium supplemented with 1% fetal bovine serum for 12h, and then treated with TGF-β1(10ng/ml) for 12, 24 and 36h.

### siRNA and Lentiviral Vectors Transduction

The RNA duplexes for siRNA-mediated ORAI1 silencing were synthesized by RiboBio (Guangzhou, China), and the sequences used were: 5’-CGTGCACAATCTCAACTCG-3’(siRNA1) and 5’-CCAGCATTGAGTGTGTACA-3’(siRNA2) as described previously (23, 31); control siRNA: 5’-TTCTCCGAACGTGTCACGT-3’. Transduction of the siRNAs(50nM) in CRC cells was performed using ribo FECT™ CP Reagent (RiboBio, Guangzhou, China) according to the manufacturer’s protocol. Plates were incubated for 48h until ready for assays. ORAI1-shRNA lentiviral vector, and scrambled-shRNA lentiviral vector were designed and generated by Neuron Biotech (Shanghai, China). Cells were seeded at 6-well plates. After incubating for 24 hours, lentiviruses were added to the cells at a MOI of 20. Culture media were changed after 24h. 72h after transfection, cells were photographed under a fluorescent microscope(NikonTE2000-U, Japan). After selection with puromycin (2 µg/ml, Beyotime, Shanghai, China) for 2 weeks, the transduced cells were used in further experiments.

### Transwell Chamber Assay

Cell migration assays were determined using the transwell chamber (8μm pore size). 5×10^4^ cells with 400µl serum-free medium were plated in the upper chamber, the culture medium (600µl) containing 10% FBS was placed in the lower chamber. After incubation for 24 h, the cells were removed from the upper chamber by a cotton swab. Then the invading cells were fixed with 4% formaldehyde and stained with 0.1% crystal violet and quantified with a light microscope. Five random fields were analyzed for each insert. Cell invasion was determined with Matrigel matrix (BD Biosciences, CA, USA) coated on the upper surface of the transwell chamber. Cells were treated as described above.

### Measurement of Calcium Entry

The store-operated Ca2+ influx was determined using the Ca2+ sensitive fluorescent indicator Rhod-2AM (AAT Bioquest Inc, CA, USA) and an inverted laser scanning confocal microscope (LEICA, TCS SP2, Germany). In brief, cells were grown on laser confocal petri dish until 70% confluent and incubated with 5 μM Rhod-2AM in Ca2+-free Hanks’ Balanced Salt Solution (HBSS)(Sangon Biotech, Shanghai, China) at 37°C for 35 min in the dark, washed twice with Ca2+-free HBSS and incubated at room temperature for 20 min. For fluorescence measurements, 5μM nifedipine (Sigma Chemical Co, MO, USA) was included in the Ca2+-free HBSS to block voltage-dependent Ca2+ channels (VDCC) (32). After 30s of baseline recording, Ca2+-free HBSS with 2μM Thapsigargin (Tg) (Sigma Chemical Co, MO, USA) was added to deplete intracellular Ca2+ store thus trigger the opening of SOCE channels and activation of SOCE. Continue scanning every 6s until cell fluorescence intensity basically returns to baseline. Then, 5mM CaCl_2_ was added to induce the Ca2+ influx. Experiments were performed for 600s in the presence of 5mM Ca2+. Fluorescence imaging was carried out at the excitation and emission wavelengths of 557nm and 581nm, respectively. In fluorescence imaging analyses, Ca2+ changes were calculated as F/F0 after background subtraction, where F is the change in fluorescence signal intensity and F0 is the baseline.

### Calpain Activation Assay

A calpain activity assay kit (Abcam, Cambridge, UK) was used to determine calpain activity in the cell according to the manufacturer’s instructions. In brief, cells were lysed in the lysis buffer at 4°C. Cell lysates were then incubated with Ac-LLY-AFC, a calpain substrate and reaction buffer for 1 h at 37°C in the dark. Fluorescence emission was measured at 505 nm using a fluorescence spectrometer with an excitation light at 400 nm.

### Immunohistochemistry (IHC)

Immunohistochemical staining was carried out on formalin-fixed paraffin-embedded blocks, and a series of sections (4-mm-thick) were prepared. Endogenous peroxidase activity was blocked by 3% hydrogen peroxide. After antigen retrieval, non-specific binding was blocked with normal goat serum. Then, the sections were incubated with rabbit anti-ORAI1 antibody (1:100, Abcam, Cambridge, UK) at 4°C overnight. Next day, the sections were incubated with secondary antibody for 45min. After the sections were counterstained, dehydrated, and mounted, the results were evaluated by two independent operators. The staining intensity was evaluated as follows: 0 (negative), 1 (weak), 2 (moderate), or 3 (strong). The staining extent was scored based on the area percentage of positive cells: 0(0-5%), 1 (6–25%), 2 (26–50%), 3 (51–75%), or 4 (>75%). The final score was the sum of staining extent and intensity. ORAI1 expression was classified as high expression (IHC score ≥4) and low expression (IHC score ≤ 3).

### Immunofluorescence Staining

Cells were grown on glass coverslips placed inside 24-well plates until 60% confluent, and then fixed with 4% formaldehyde for 15 min at room temperature, After washing, Immunostaining Permeabilization Solution with Saponin (Beyotime Biotech, Shanghai, China) was added and incubated for approximately 5min, and then blocked with 4% BSA for 1 h. Anti-E-cadherin(1:200, Santa Cruz, CA, USA) or anti-Vimentin (1:200, Santa Cruz, CA, USA) primary antibody was incubated with blocked cells overnight at 4°C. After removing the primary antibodies and washing with PBS for three times, cells were incubated with Cy3-conjugated secondary antibody (1:300, Proteintech, Wuhan, China) for 1h at room temperature, followed by incubation with DAPI (Sangon Biotech, Shanghai, China) for 5min. After washing with PBS, coverslips were sealed with anti-quenching fluorescent mounting medium. Images were captured using a fluorescence microscope (Nikon TE2000-U, Japan).

### Quantitative RT-PCR

Total RNA was isolated using the Trizol reagent (Invitrogen, CA, USA) and cDNA was generated using the PrimeScript™ RT reagent Kit (Takara, Dalian, China). Quantitative real-time PCR was performed with SYBR Premix Ex Taq (Takara, Dalian, China) using CFX96 Real Time PCR System (Bio-Rad, CA, USA). The RT primers were as follows: ORAI1: forward 5’-GCTGCTCTGCTGGGTCAAGTT-3’and reverse 5’- CGATAAAGATCAGGCCGAAGG-3’, GAPDH: forward 5’-CTTTGGTATCGTGGAAGGACTC-3’ and reverse 5’- GTAGAGGCAGGGATGATGTTCT -3’. The cycling program was: Pre−denaturation at 95˚C for 30 seconds, followed by 40 cycles of 95°C for 5 seconds, 60°C for 30 seconds, and extension at 65˚C for 1min. Relative expression level of ORAI1 was normalized to GAPDH and calculated using the2^−ΔΔCt^ method.

### Western Blot

The tissue samples and cells were lysed using RIPA Lysis Buffer (Beyotime Biotech, Shanghai, China) containing PMSF (Beyotime Biotech, Shanghai, China). The protein concentration was detected using the BCA kits. Equal amounts of protein were separated by 10%SDS-PAGE and transferred onto polyvinylidene difluoride membranes. After blocking with 5% nonfat milk in Tris-buffered saline-Tween (TBST) for 2h at room temperature, membranes were incubated with primary antibodies against ORAI1(1:500, Proteintech, Wuhan, China), E-cadherin (1:1000, Santa Cruz, CA, USA), N-cadherin (1:1000, Cell Signaling Technology, MA, USA)), Vimentin (1:1000, Santa Cruz, CA, USA), β-actin (1:2000, Proteintech, Wuhan, China) overnight at 4°C. After washing with TBST, membranes were incubated with HRP-labeled secondary antibodies for 2h at room temperature. Protein bands were detected using an enhanced chemiluminescence detection kit (ECL). Densitometric analysis was performed using Fusion software (Vilber Lourmat, France). β-actin was used as loading control.

### Statistical Analysis

All experiments were repeated three times and all of the data were analyzed by SPSS19.0 software. Measurement data are expressed as mean ± standard deviation (SD). Comparison was made by the Student’s *t* test between two groups and by the one-way ANOVA among multiple groups. The *χ* ^2^ test was used to analyze the correlation between ORAI1 expression and clinicopathologic parameters. The survival curves were generated by Kaplan-Meier method and analyzed by the log-rank test. *P*<0.05 was considered statistically significant.

## Results

### ORAI1 Is Highly Expressed in Human CRC Tissues and Indicates Poor Clinical Outcomes

**Figure 1:**
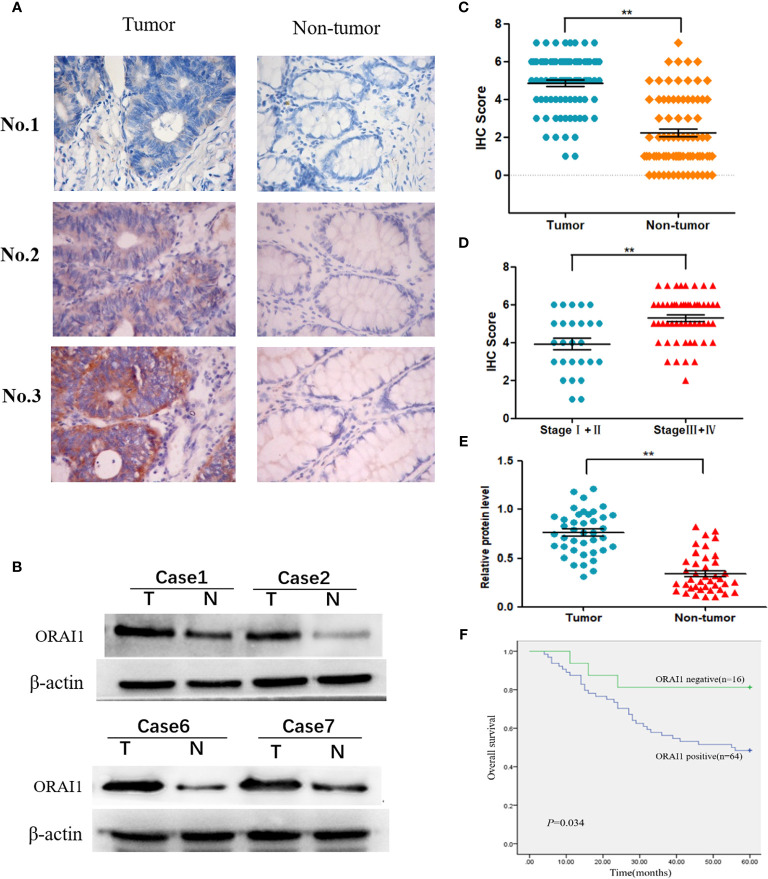
Expression of ORAI1 is significantly upregulated in CRC tissues. **(A)** Representative immunohistochemistry staining of ORAI1 expression in 80 paired CRC tissues. magnification, ×400. **(B)** Western blot was applied to detect protein expression levels of ORAI1 in paired CRC tumor tissues (T) and non-tumor tissues(N). **(C)** Immunohistochemistry average staining scores of tumor tissues and matched non-tumor tissues. ^**^P<0.01. **(D)** IHC average staining scores of tumor tissues in stage I+II and stage III+IV patients. ^**^P<0.01. **(E)** The protein relative expression levels of ORAI1 in tumor tissues and non-tumor tissues of 40 patients by Western blot. ^**^P<0.01. **(F)** Kaplan–Meier’s analysis of the correlation between ORAI1expression and overall survival (OS) of CRC patients.

To investigate the expression of ORAI1 in human CRC tissues, we first examined ORAI1 expression in 80 CRC tissue samples and non-tumor tissues by immunohistochemical staining (IHC). As shown in **Figure 1A**, the results of IHC revealed that ORAI1 was mainly expressed in the membrane and cytoplasm. High expression (IHC score≥4) was assessed in 64 of 80 (80%) primary CRC samples compared with only in 24 of 80 (30%) paired non-tumor tissues, average staining scores of 4.85 and 2.23, respectively (**Table 1**, **Figure 1C**). Furthermore, ORAI1 expression was significantly higher in stage III and IV patients than that in stage I and II patients (5.30 vs. 3.92) (**Figure 1D**). We further detected ORAI1 expression in an additional 40 fresh CRC tissues and the corresponding non-tumor tissues by Western blot analysis. Consistent with IHC observations, ORAI1 was expressed at higher levels in CRC tumors compared with matched adjacent non-tumor tissues (**Figures 1B, E**).

**Table 1:** Upregulation of ORAI1 in CRC tissues.

ORAI1 staining	Tumor	Adjacent non-tumor	*χ^2^*	*P*
Positive	64	24	40.4	<0.01
Negative	16	56		

Furthermore, we analyzed the association between ORAI1 expression and clinicopathological parameters in 80 CRC patients by IHC staining (**Table 2**). The results showed that the patients with high ORAI1 expression had a higher tendency to be with advanced clinical stage, high incidence of metastasis. But there were no statistical connections between ORAI1 overexpression and age, gender, and tumor size. Finally, we analyzed the association between ORAI1 expression and survival of CRC patients. By Kaplan-Meier survival analysis, we found that patients with higher ORAI1 expression were likely to be with significantly shorter overall survival (**Figure 1F**). Taken together, these results suggest that high expression of ORAI1 is associated with tumor progression and poor clinical outcomes.

**Table 2:** Correlation between ORAI1 and clinical parameters of CRC patients.

Variables	Case	ORAI1		*χ^2^*	*P*
		-	+		
Age (years)					
≥60	32	6	26	0.052	0.819
<60	48	10	38		
Gender					
Male	55	10	45	0.364	0.546
Female	25	6	19		
Tumor size (cm)					
≥5	33	4	29	2.179	0.140
<5	47	12	35		
TNM stag					
I-II	26	11	15	11.980	0.001^**^
III-IV	54	5	49		
Lymph node metastasis (N_2_)					
Yes	31	2	29	5.806	0.016^*^
No	49	14	35		

*P<0.05; **P<0.01.

### Expression of ORAI1 Is Different in Primary (SW480) and Metastatic (SW620) Colon Cancer Cell Lines

**Figure 2:**
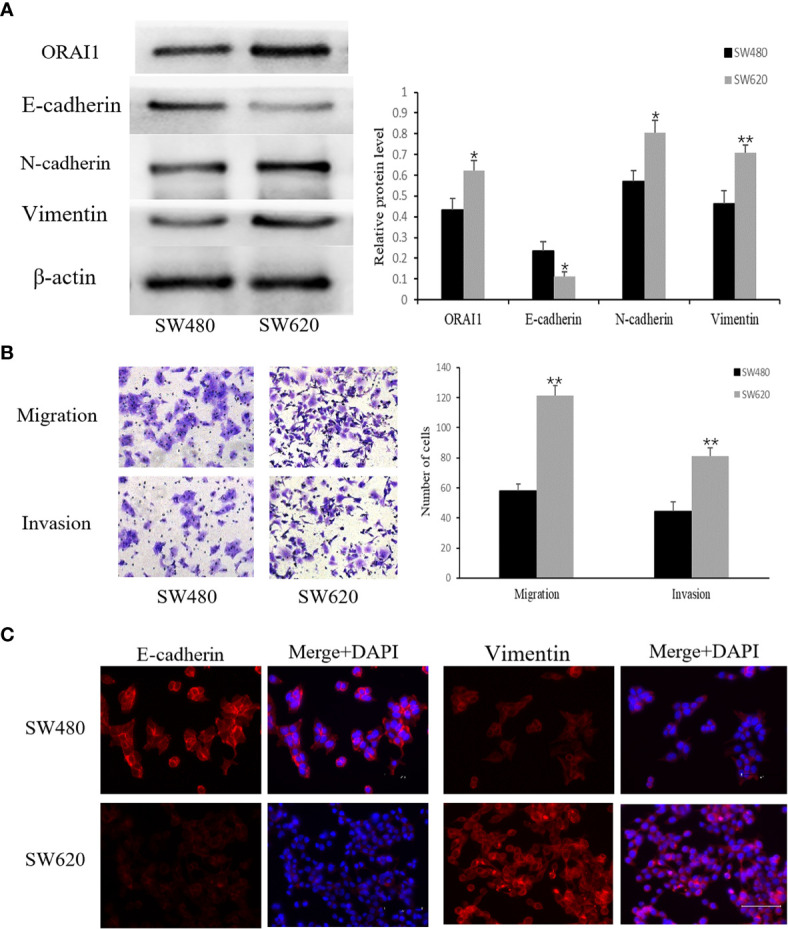
SW480 and SW620 cells have different expression levels of ORAI1 and EMT-relevant markers. **(A)** Western blot analysis of ORAI1 and EMT-relevant markers expression in these two CRC cell lines. **(B)** The metastatic capability of these two cell lines was detected by transwell migration and invasion assays. magnification, ×200. **(C)** Immunofluorescence was performed to evaluate the expression of E-cadherin and Vimentin in these two CRC cell lines, Scale bar 75μm. All the experiments were repeated three times. *P<0.05; **P<0.01.

Having identified the expression properties of ORAI1 in human CRC tissues, we further evaluated its expression level in two human CRC cell lines SW480 and SW620. Interestingly, Western blot analysis showed ORAI1 expression was significantly higher in SW620 cells than in SW480 cells (**Figure 2A**). The SW480 cell line was obtained from a primary CRC lesion, and the SW620 cell line was established from a lymph node (LN) metastasis in the same patient a year later, which exhibited higher metastasis potential (33). Therefore, the two cell lines have the same genetic background but different metastatic potential. Transwell assay also showed that SW620 cells migrate and invade more aggressively than SW480 cells (**Figure 2B**).

Importantly, a key feature of cancer metastasis is the initiation of EMT. Therefore, we detected expression of EMT-relevant markers in the two cell lines. As expected, expression of E-cadherin, an epithelial marker, was substantially lower in SW620 cells than in SW480 cells, but a significantly higher expression of Vimentin and N-cadherin, two mesenchymal markers, was observed in SW620 cells (**Figure 2A**). These changes in E-cadherin and Vimentin expression were confirmed by an immunofluorescent assay (**Figure 2C**). Combined with the distinct migratory ability, expression levels of EMT-related markers and ORAI1 between the two cell lines, these results suggest that ORAI1 might be involved in the EMT-derived migration in CRC cells.

### Knockdown of ORAI1 Suppresses the EMT of SW620 Cells

**Figure 3:**
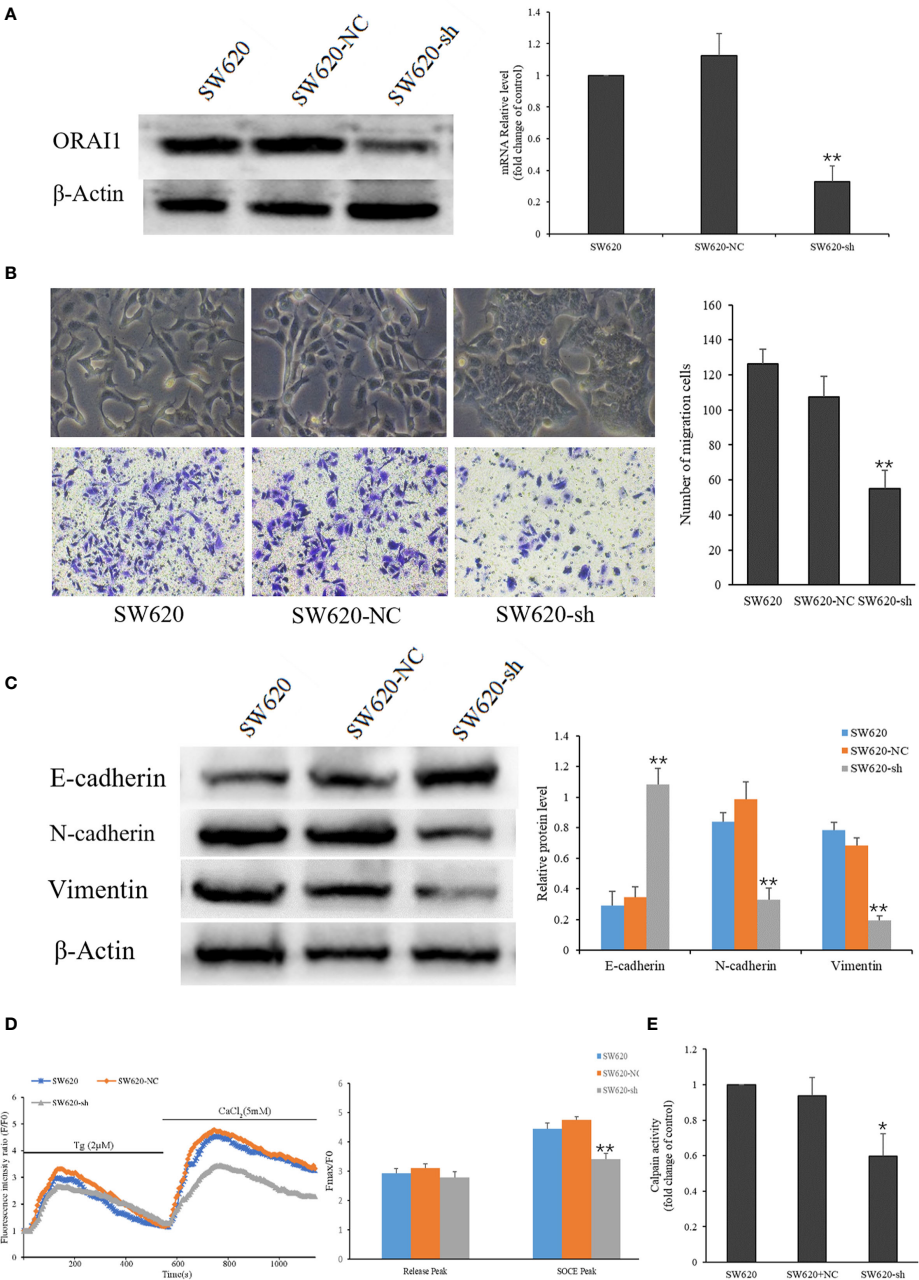
ORAI1 knockdown suppresses the EMT of SW620 Cells. **(A)** SW620 cells were infected with lentivirus containing sh-ORAI1 or control sh-RNA lentivirus (NC) for 72 hours. Knockdown efficiency was confirmed by QRT-PCR (right panel) and Western blot (left panel). **(B)** Representative picture showed the morphological change of SW620 cells after infecting with lentivirus for 72 h. Cell migration was assessed by transwell assay. Magnification, ×200. **(C)** The protein levels of E-cadherin, N-cadherin and Vimentin were analyzed by Western blot. **(D)** Representative traces of intracellular Ca2+ signals were assessed by Rhod-2AM Ca2+ measurement. **(E)** Quantitative assessment of calpain activity in SW620 and the transfected cells. All the experiments were repeated three times. *P<0.05; **P<0.01.

As mentioned above, the SW620 cell line was obtained from a CRC lymph node (LN) metastasis, which exhibited higher EMT characteristics. To investigate the role of ORAI1 in the EMT of SW620 cells, SW620 cells were transduced with two ORAI1-specific siRNAs and a control siRNA, respectively, and we found that SW620-siRNA1 and SW620-siRNA2 cells exhibited 87% and 84% decrease in protein levels and 69% and 63% in mRNA levels (**Supplementary Figure S1A**). Transwell assays also showed that transduction with siRNA1 or siRNA2 in SW620 cells markedly decreased cell migration (**Supplementary Figure S1C**). Western blot analysis showed that both siRNA1 and siRNA2 transduction could suppress the EMT of SW620 cells(**Supplementary Figure S1D**). Given that siRNA1 has a slightly better knock-down effect, it was therefore selected as the target sequence for subsequent experiments. Accordingly, we used this sequence to construct the ORAI1-shRNA lentiviral vectors, and the expression of ORAI1 in the transfected cells was confirmed by QRT-PCR and Western blot. The results showed that cells transfected with ORAI1 shRNA exhibited 82% decrease in protein levels and 67% in mRNA levels (**Figure 3A**). By comparing the morphology of the cells under a light microscope, we observed that cells with ORAI1 shRNA showed highly organized cell-cell adhesion and cobblestone shape with epithelioid morphology appeared (**Figure 3B**). To further explore cell migration capacity after ORAI1 silencing, transwell assay was performed subsequently. The results showed the number of migrated cells was markedly decreased in ORAI1 silence cells compared to the negative control (**Figure 3B**).

Next, we assessed the effect of ORAI1 knockdown on the EMT-relevant molecules in these cell models. Western blot analysis showed that the expression of E-cadherin was significantly up-regulated, and the expression of N-cadherin and Vimentin was obviously down-regulated in SW620-shORAI1 cells, compared with control cells (**Figure 3C**). As ORAI1 is an essential component of SOCE channel, we further investigated SOCE initiation in these cells. When the medium was absent of Ca2+, addition of thapsigargin, an ER Ca2+-ATPase inhibitor, resulted in a rapid rise in intracellular Ca2+ level, consistent with depletion of ER Ca2+ stores. Subsequently, addition of Ca2+, which initiates Ca2+ influx, triggered another significant increase in the Ca2+ level (SOCE). As shown in **Figure 3D**, knockdown of ORAI1 decreased Ca2+ entry in SW620-shORAI1 cells compared with control cells. Following the inhibition of SOCE, decrease of the calpain activity was also observed in SW620-shORAI1 cells (**Figure 3E**). These results suggest that ORAI1 plays an important role in the acquisition of EMT in CRC cells. To further investigate the mechanism of ORAI1 in TGF-β induced EMT in CRC cells, we chose the SW480 cell line, which is characterized by a modest more mesenchymal characteristics, for the subsequent experiment.

### TGF-β1 Induces EMT and Enhances ORAI1 Expression in SW480 Cells

**Figure 4:**
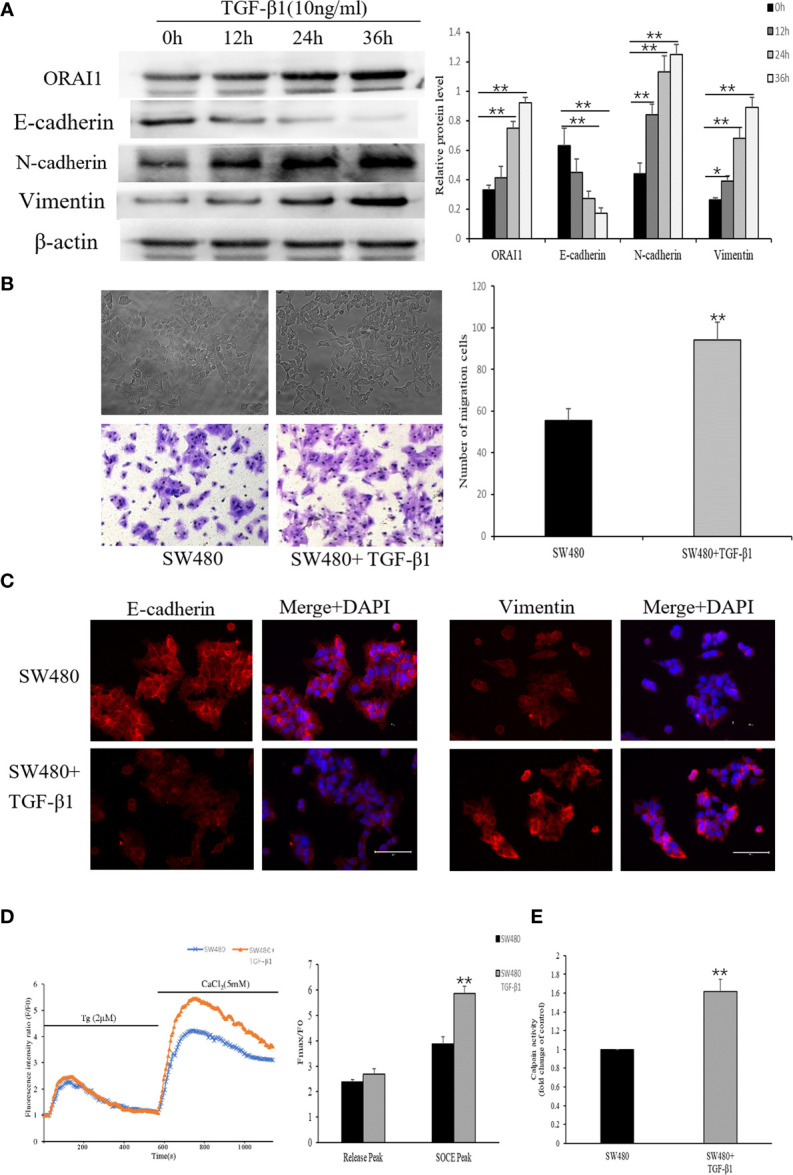
TGF-β1 induces EMT and increases ORAI1-mediated Ca2+ entry in SW480 cells. **(A)** Western blot was performed to detect the expression of ORAI1 and EMT-relevant markers in SW480 cells treated with 10ng/ml TGF-β1 for 0,12,24 and 36h. **(B)** Representative picture showed the morphological change of SW480 cells after treatment with 10ng/ml TGF-β1 for 24 h. Cell migration was assessed by transwell assay. Magnification, ×200. **(C)** Immunofluorescence was performed to evaluate the expression of E-cadherin and Vimentin in SW480 cells after treatment with TGF-β1, Scale bar 75μm. **(D)** Representative traces of intracellular Ca2+ signals of SW480 cells with or without TGF-β1 treatment were assessed by Rhod-2AM Ca2+ measurement. **(E)** Quantitative assessment of calpain activity. All the experiments were repeated three times. *P<0.05; **P<0.01.

Previous studies suggested that TGF-β1 is a powerful EMT stimulus (34), which is known to induce tumor cell invasion by activating EMT in metastatic cancer thereby enhancing invasiveness and metastasis (35). For this, we induced the EMT process in SW480 cells by treatment with 10ng/ml TGF-β1 for 0, 12, 24 or 36h. Time course responses to TGF-β1 were tested as well on the expression of E-cadherin, N-cadherin and Vimentin protein. Western blot analysis confirmed significant decreases in the levels of the E-cadherin protein and increases in the expression of Vimentin and N-cadherin protein in SW480 cells that had been treated with 10ng/ml TGF-β1 for 24 or 36 h (**Figure 4A**). These results suggest that TGF-β1 induces EMT most significantly after 24 h treatments in SW480 cells. Therefore, cells were incubated with 10ng/ml TGF-β1 for 24h in subsequent experiments.

By comparing the morphology of the cells under a light microscope, we observed that cells stimulated with TGF-β1 became an elongated morphology with losing polarity and cell–cell contacts (**Figure 4B**). Next, we further confirmed the effects of TGF-β1 on EMT in SW480 cells, using transwell assay and immunofluorescence. Transwell assay analysis showed that the number of cells migrated indeed had a significant increase in the migration of SW480 cells treated with TGF-β1 for 24h (**Figure 4B**). Based on the immunofluorescence staining for EMT-related molecules, E-cadherin expression was decreased and Vimentin expression was increased in TGF-β1-treated cells (**Figure 4C**). TGF-β has also been shown to regulate Ca2+ signaling (29), and thus we investigated whether store-operated Ca2+ entry (SOCE) initiation is altered upon treatment with TGF-β1. As shown in **Figure 4D**, SOCE activity was significantly increased in TGF-β1 treatment cells. Following the increased Ca2+ influx, overall calpain activity was measured, which showed a significant increase in SW480 cells after TGF−β1 treatment (**Figure 4E**). We then assessed whether TGF-β1 promotes Ca2+ influx accompanied by up-regulation of ORAI1 expression. Interestingly, Western blot analysis showed the expression of ORAI1 also increased with TGF-β1 treatment for 24 or 36h (**Figure 4A**). These results presented above suggest that TGF-β1-induced activation of ORAI1 and SOCE could be crucial for promoting EMT.

### Knockdown of ORAI1 Attenuates the TGF-β1-Induced EMT in SW480 Cells

**Figure 5:**
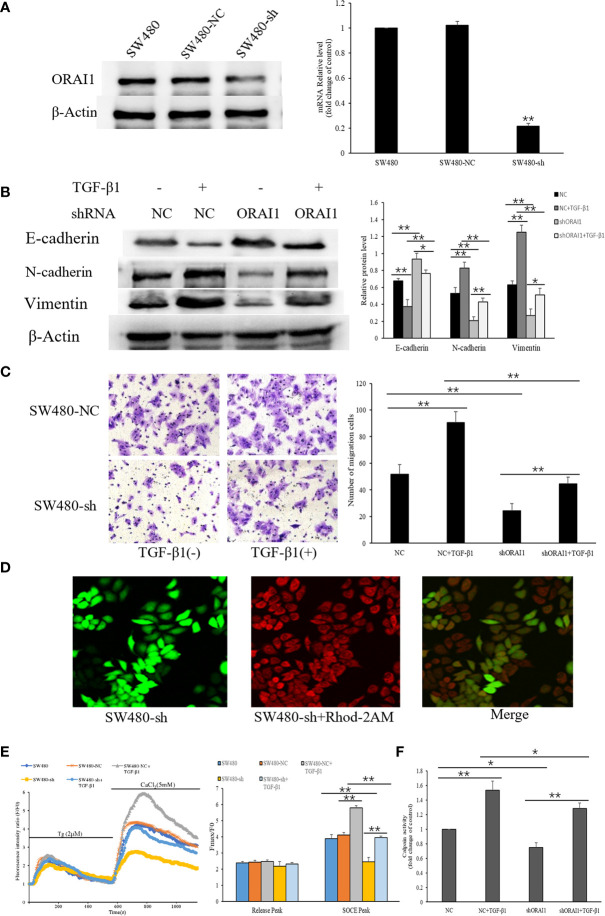
ORAI1 silencing attenuates the TGF-β1-induced EMT in SW480 cells. **(A)** SW480 cells were infected with lentivirus containing shORAI1 or control shRNA lentivirus (NC) for 72 hours. Knockdown efficiency was confirmed by QRT-PCR (right panel) and Western blot (left panel). **(B)** SW480-shORAI1 cells and SW480-NC cells were stimulated with or without TGF-β1(10ng/ml) for 24h, and the protein levels of E-cadherin, N-cadherin and Vimentin were analyzed by Western blot. **(C)** Migration capacity of SW480-shORAI1 cells and SW480-NC cells was assessed by transwell assay. Magnification, ×200. **(D)** SW480 cells were treated with the Ca2+ sensitive fluorescent indicator Rhod-2AM, and fluorescence signals were detected by an inverted laser scanning confocal microscope, Scale bar 75μm. **(E)** Representative traces of intracellular Ca2+ signals were assessed by Rhod-2AM Ca2+ measurement. **(F)** Quantitative assessment of calpain activity in SW480-shORAI1 and SW480-NC cells with or without TGF-β1 treatment. All the experiments were repeated three times. *P<0.05; **P<0.01.

To verify whether ORAI1 is responsible for TGF-β1-induced EMT, SW480 cells were transduced with the two ORAI1-specific siRNAs. The knockdown efficiency was confirmed by QRT-PCR and Western blot, and we found that SW480-siRNA1 and SW480-siRNA2 cells showed 78% and 72% decrease in protein levels and 81% and 76% in mRNA levels (**Supplementary Figure S1B**). Transwell assays also showed that the number of migrated cells was significant reduction in SW480-siRNA1 or SW480-siRNA2 cells (**Supplementary Figure S1C**). Western blot analysis also showed that similar changes of EMT-relevant marks expression were found in these cells (**Supplementary Figure S1E**). These results indicated that siRNA1 also exhibited a slightly better knock-down effect in SW480 cells. Therefore, SW480 cells were infected with lentiviral vector expressing ORAI1 shRNA or a control shRNA lentivirus (NC), and we found that 61% decrease in protein levels and 79% in mRNA levels in SW480-shORAI1 cells, and without changes in SW480-NC cells (**Figure 5A**). These results suggest that we successfully silenced ORAI1 expression in SW480 cells with ORAI1-targeted shRNA. First, EMT-relevant markers were evaluated by Western blot. We found that ORAI1 knockdown induced a significant up-regulation of E-cadherin expression in parallel to a significant down-regulation of the N-cadherin and Vimentin expression, and ORAI1 downregulation could rescue E-cadherin inhibition after TGF−β1 treatment and alleviate the increase of N-cadherin and Vimentin expression induced by TGF-β1 (**Figure 5B**). To further establish that cell migration is also affected upon ORAI1 silencing, transwell assay was performed. The results showed the number of migrated cells was clearly decreased for ORAI1 silence cells, and knockdown of ORAI1 significantly rescinded the increase in TGF-β1-induced migration (**Figure 5C**).

As ORAI1 can induce Ca2+ entry through SOCE mechanisms, we further detected SOCE initiation in SW480-shORAI1 cells and SW480-NC cells that were treated or not treated with TGF-β1 (**Figures 5D, E**). As shown in **Figure 5E**, TGF−β1 treatment also significantly increased Ca2+ entry in SW480-NC cells. Knockdown of ORAI1 not only decreased Ca2+ entry in SW480-shORAI1 cells, but also significantly attenuated TGF-β1-evoked Ca2+ entry compared with SW480-NC cells. Next we examined how a reduction in TGF-β1-induced activation of Ca2+ entry by shORAI1 affected Ca2+-dependent calpain activity. Decrease of the basal calpain activity levels was observed in SW480-shORAI1 cells, and silencing of ORAI1 abolished the increased calpain activity seen with TGF-β1 treatment (**Figure 5F**). These results strongly suggest ORAI1 downregulation was associated with a decrease of SOCE, and ORAI1 and ORAI1-mediated SOCE is involved in regulating TGF-β1-induced EMT.

### Blockade of SOCE Alleviates the TGF-β1-Induced EMT in SW480 Cells

**Figure 6:**
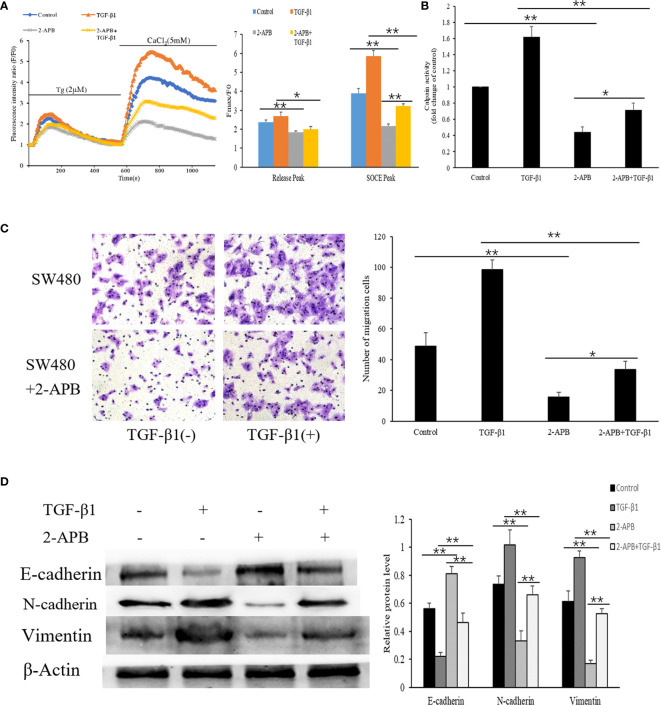
Blockade of SOCE alleviates the TGF-β1-induced EMT in SW480 cells. **(A)** Representative traces of intracellular Ca2+ signals of SW480 cells with 2-APB and/or TGF-β1treatment were assessed by Rhod-2AM Ca2+ measurement. **(B)** Quantitative assessment of calpain activity in SW480 cells treated with 2-APB and/or TGF-β1. **(C)** SW480 cells were treated with 2-APB and/or TGF-β1, and cell migration was assessed by transwell assay. magnification, ×200. **(D)** Protein levels of E-cadherin, N-cadherin and Vimentin were analyzed by Western blot in SW480 pretreatment cells. All the experiments were repeated three times. *P<0.05; **P<0.01.

The results mentioned above indicated that the effect of TGF-β1 on EMT may be related to increased SOCE-related Ca2+ influx. We therefore evaluated whether blocking store-mediated Ca2+ entry with 2-APB (2-Aminoethyl diphenylborinate), a pharmacologic inhibitor which has been previously shown to block calcium entry through ORAI channels (7), can alter the effects of TGF-β1. SW480 cells pretreated with or without TGF-β1were treated with 2-APB(100µM) for 30min. Ca2+ entry, transwell migration assay, and EMT-relevant markers were detected. As shown in **Figure 6A**, we found that cells treated with 2-APB showed a significant reduction in Ca2+ release (addition of thapsigargin) and Ca2+ entry (addition of Ca2+). Interestingly, a dramatic decrease in Ca2+ entry and Ca2+ release were also observed in the cells treated with TGF-β1 and 2-APB compared with TGF-β1 treatment cells. According to the calpain activity assays, treatment of 2-APB also repressed the TGF-β1-mediated Ca2+–dependent calpain activity (**Figure 6B**).

As blockade of SOCE has been previously shown to prevent cellular migration associated with EMT (36), to establish that cell migration is also affected upon Ca2+ influx in SW480 cells, transwell migration assay was performed which showed that treatment of 2-APB significantly inhibited cell migration in SW480 cells. Similarly, the increased migration seen with TGF-β1 treatment was attenuated by a blockage of Ca2+ entry with 2-APB (**Figure 6C**). Next, we further evaluated the effect of 2-APB treatment on EMT-relevant markers by Western blot. The results showed that 2-APB treatment of SW480 cells resulted in a pronounced increase in E-cadherin expression and a decrease in N-cadherin and Vimentin. Consistent with these results, TGF-β1-induced decrease in E-cadherin expression and increase in N-cadherin and Vimentin were also abolished by the addition of 2-APB (**Figure 6D**). Taken together, these results suggest that SOCE-related Ca2+ influx also contributes to the EMT induced by TGF-β1 in SW480 cells. In addition, 2-APB and sh-ORAI1 both exhibited similar inhibition effects to the SW480 cells.

## Discussion

According to the latest data from the American Cancer Society, CRC is the third most common cause of cancer death in both men and women in the United States, and ranks second when men and women are combined (37). Mortality from CRC is mostly due to invasion and metastasis of neoplastic cells from the primary tumors to distant organ sites (29). Hence, understanding the mechanism regulating metastasis is critical to improving colon cancer survival. EMT is an important biological hallmark for epithelial cancer (38). Although EMT was first noticed during embryonic development, it is increasingly acknowledged that EMT makes a greater contribution to the tumor progression by enhancing migratory and invasive capabilities of cancer cells (39). During EMT processes, epithelial cells lose the expression of the epithelial characteristic marker, like E-cadherin and acquire the expression of the mesenchymal cell marker, such as Vimentin (40). This process has been shown to be associated with the Ca2+ influx (10). Ca2+ has versatility as a second messenger and it regulates many cellular functions by influencing various genes, proteins and signaling pathways (21). It regulates intracellular enzyme activity and protein-protein interactions through calmodulin or other calcium-binding proteins (26). Many types of cancer have been shown to have dysregulated expression of Ca2+ channels and other molecules involved in Ca2+ homeostasis (29). But the role of Ca2+ in EMT-associated metastasis is still not well understood. Ca2+ entry into tumor cells has been confirmed to be due to SOCE mediated by ORAI1 channels and this process in malignant tumors have fascinated many investigators, and thus the channels were already designated to “onco-channels” (21, 41). ORAI1-mediated SOCE has been confirmed to regulate cancer cell proliferation and invasion (24), and it has been reported that ORAI1 has an effect on CRC cell proliferation (22), but the research on the role of ORAI1-mediated SOCE in CRC is limited. Therefore, effects of ORAI1 on CRC should be warranted to investigate.

Emerging role of ORAI1 in human cancer has been reported. Upregulation of ORAI1 expression was observed in multiple human cancers, including non-small cell lung carcinoma (19), esophageal cancer (42) and gastric cancer (20). Consistent with these observations, we found that ORAI1 expression was markedly upregulated in CRC tissues compared with matched adjacent non-tumor tissues in this study. Moreover, we further examined the clinical relevance of ORAI1, and found that high expression of ORAI1 was closely associated with advanced clinical stage, high incidence of metastasis and shorter overall survival times. A previous study agreed with these conclusions demonstrating that ORAI1 overexpression was significantly correlated with poor prognosis in breast cancer (27). These results suggested that ORAI1 may be involved in CRC progression. In addition, since different cancer cell lines have distinct gene background, the genes that impact tumor progression may be differently expressed in these cells (43). Therefore, we further evaluated ORAI1 expression level in two human CRC cell lines SW480 and SW620, which have the same genetic background but different metastatic potential. In the present study, we provided evidence that ORAI1 expression differed in the two CRC cancer cell lines: SW620 cells, with more mesenchymal like characteristics and higher metastasis potential, express ORAI1 at higher levels than the SW480 cells with modest more epithelial characteristics. A recent study also demonstrated that STIM1 and ORAI3 are upregulated in metastatic CRC cells compared to primary CRC cells (44). Moreover, we also found that knockdown of ORAI1 suppressed the EMT of SW620 cells. These results suggest that ORAI1 might be involved in the EMT of CRC cells.

TGF-β1 is one of the most abundant growth factors stored and released by bone (45). Previous studies indicated that TGF-β1 can promote cancer cell invasion by regulating the induction of EMT (37). In our studies, when incubated with TGF-β1, SW480 cells underwent morphological changes from an “epithelial” polarized morphology to a mesenchymal fibroblastoid morphology and achieved increased migratory capabilities. This phenotypic transformation was accompanied by down-regulation of epithelial signature proteins including E−cadherin and increased expression of mesenchymal markers such as E−cadherin and Vimentin. However, the underlying mechanisms for TGF-β1 induced EMT of CRC cells are unclear. Previous studies demonstrated calcium signaling plays an important role in TGF-β signaling (46), and different mechanisms have been suggested to be involved in the regulation of Ca2+ homeostasis by TGF-β1 (38). Our studies show that TGF-β1 treatment enhanced the expression of ORAI1, an essential component of SOCE channel and promotes SOCE-related Ca2+ influx in SW480 cells. This result was similar with the result from a previous research that TGF-β1-induced EMT significantly increased SOCE in MCF7 breast cancer cells (9). Ca2+-dependent calpain contributes to many pathways that control cell migration, and M-calpain activity was shown to be significantly increased in CRC (47). In the present study, we show that TGF-β1 treatment amplified the calpain activity. These results provide further evidence for the importance of ORAI1, SOCE and calpain activity in TGF-β1-induced EMT of CRC cells.

To further verify the role of ORAI1 and ORAI1-mediated SOCE is critical for TGF-β1-induced EMT of CRC cells, we examined the effects of pharmacological inhibition for SOCE or gene silencing of ORAI1. 2-APB, a nonspecific pharmacological inhibitor, was previously shown to block SOCE through ORAI1 channels (48). Interestingly, blockade of SOCE markedly mitigated TGF-β1-induced migration and prevented reduced expression of E−cadherin protein after TGF−β1 treatment. Meanwhile, our results also showed that TGF-β1-induced increases in N-cadherin and Vimentin were decreased upon inhibition of Ca2+ influx by 2-APB, both of which are major cytoskeletal component of mesenchymal cells and necessary for motility seen in EMT. In addition, genetic studies using silencing method further established that ORAI1 is the major Ca2+ homeostasis channel in CRC cells. Our data confirmed that knockdown of ORAI1 led to decrease in Ca2+ influx, and significantly suppressed cell migration and changes of EMT-relevant marks expression induced by TGF-β1. This finding is consistent with the results of a previous study reporting that knockdown of ORAI1 in glioma can significantly reduce receptor-dependent Ca2+ influx and inhibit Pyke activity, thereby inhibiting glioma cell invasion and metastasis (49). Parallel phenomena were also observed for calpain activity. Importantly, a decrease in calpain activity was observed when Ca2+ influx was inhibited by 2-APB. Furthermore, knockdown of ORAI1 or inhibition of SOCE inhibited TGF-β1-induced increase in calpain activation. These results indicate that ORAI1 and ORAI1-mediated SOCE is involved in TGF-β1–induced EMT in SW480 cells.

## Conclusion

In conclusion, our study indicated that ORAI1 expression was significantly upregulated in CRC, and ORAI1 overexpression was correlated with poor prognosis. we also had demonstrated that ORAI1 knockdown suppressed the EMT of SW620 Cells, and TGF-β1 treatment induced the EMT process in SW480 cells, increased ORAI1 expression, SOCE-mediated Ca2+ entry and calpain activity. Meanwhile, the effect of TGF-β1 treatment was obviously abolished by inhibiting Ca2+ influx with 2-APB and ORAI1 knockdown. Therefore, it can be inferred that TGF-β1induces EMT of SW480 cells by enhancing ORAI1 expression, promoting ORAI1-mediated Ca2+ entry and calpain activity. This is a possible mechanism for the TGF-β1-induced EMT in SW480 cells. Hence, ORAI1-mediated Ca2+ signaling may serve as a potential therapeutic target for CRC.

## Data Availability Statement

The original contributions presented in the study are included in the article/**Supplementary Material**. Further inquiries can be directed to the corresponding author.

## Ethics Statement

The studies involving human participants were reviewed and approved by Medical Ethics Review Committee of the First Affiliated Hospital of Chongqing Medical University. The patients/participants provided their written informed consent to participate in this study.

## Author Contributions

QK, BY, and ZW designed the study. QK and BY performed the main experiments. DH and GW collected patient specimens. XP and XL analyzed the data. QK wrote the manuscript. All authors contributed to the article and approved the submitted version.

## Funding

This study was supported by Chongqing key diseases Research and Application Demonstration Program (Colorectal Cancer Prevention and Treatment Technology Research and Application Demonstration [No. 2019ZX003]).

## Conflict of Interest

The authors declare that the research was conducted in the absence of any commercial or financial relationships that could be construed as a potential conflict of interest.
