# Supplementary material for: Calcium Channel Protein ORAI1 Mediates TGF-β Induced Epithelial-to-Mesenchymal Transition in Colorectal Cancer Cells
Source: Front Oncol. 2021 May 12;11:649476. doi: 10.3389/fonc.2021.649476 (PMC8149897; doi:10.3389/fonc.2021.649476)
Supplement: Supplementary file 1 [file DataSheet_1.docx]

Fig. S1


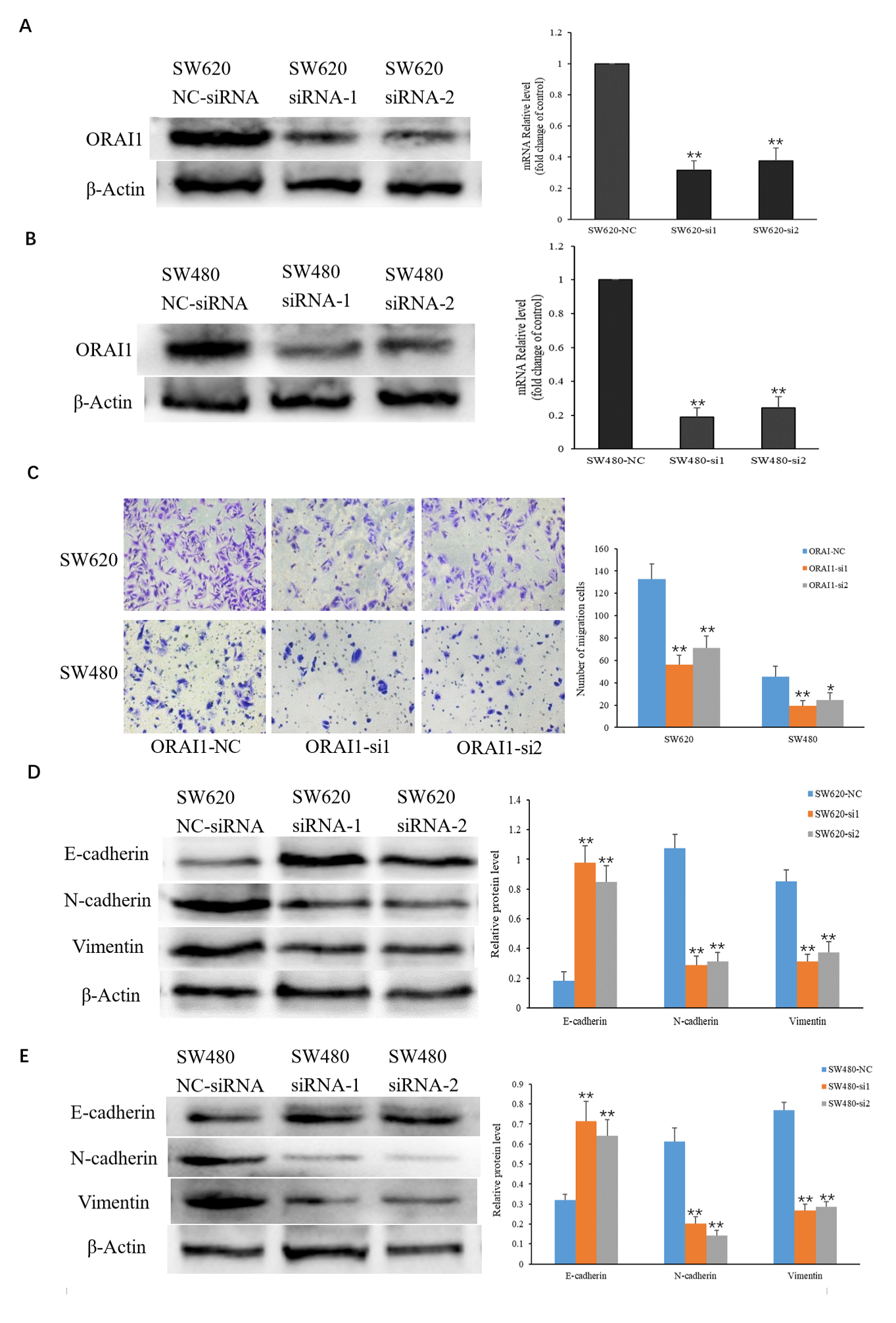


**FIGURE. S1 The inhibition effect of ORAI1-siRNAs** in SW620 and SW480 cells. **(A)** The effectiveness of two ORAI1-siRNAs in knocking-down the expression level of ORAI1 in SW620 cells was confirmed by QRT-PCR (right panel) and Western blot (left panel). **(B)** Knockdown efficiency of the two ORAI1-siRNAs in SW480 cells was confirmed by QRT-PCR (right panel) and Western blot (left panel). **(C)** Cell migration was assessed by transwell assay. Magnification, ×200. **(D)**The protein levels of E-cadherin, N-cadherin and Vimentin were analyzed by Western blot in SW620 transduced cells. **(E)** The protein levels of E-cadherin, N-cadherin and Vimentin were analyzed by Western blot in SW480 transduced cells. All the experiments were repeated three times. **P*<0.05; ***P*<0.01
